# Non-Invasive Pregnancy Diagnosis in Big Cats using the PGFM (13,14-dihydro-15-keto-PGF_2α_) Assay

**DOI:** 10.1371/journal.pone.0143958

**Published:** 2015-12-03

**Authors:** Martin Dehnhard, Vinod Kumar, Mithileshwari Chandrasekhar, Katarina Jewgenow, Govindhaswamy Umapathy

**Affiliations:** 1 Department of Reproduction Biology, Leibniz Institute for Zoo & Wildlife Research (IZW), 10315, Berlin, Germany; 2 CSIR-Laboratory for the Conservation of Endangered Species, Centre for Cellular and Molecular Biology, Uppal Road, Hyderabad, 500007, India; Oregon State University, UNITED STATES

## Abstract

Non-invasive monitoring of hormones using feces has become a vital tool for reproductive management and reliable pregnancy diagnosis in big cats. Previous studies described the PGF_2α_ metabolite (PGFM) as an indicator of pregnancy in various feline species. The present study aimed to standardize pregnancy detection in big cats like the tiger (*Panthera tigris)*, jaguar (*Panthera onca)* and lion (*Panthera leo)* using fecal samples. High-performance liquid chromatography (HPLC) and liquid chromatography-mass spectrometry (LCMS) were performed to identify PGFM in feces. An EIA developed against 9α,11α-dihydroxy-15-oxo-prost-5-en-1-oic acid-BSA was used to assay PGFM in fecal samples of the Bengal tiger, Asiatic lion and jaguar. The PGFM levels increased after 9 weeks of pregnancy and remained elevated until parturition. All animals showed elevated levels of PGFM in the last trimester of pregnancy, thus making PGFM a reliable tool for pregnancy diagnosis during this period that can be useful in captive breeding programs in these species.

## Introduction

About 36 feline species are found around the world and many of them are listed in the IUCN Red List as vulnerable or endangered due to poaching for trade, to habitat loss and fragmentation of natural habitats. Most of the big cats such as lions, tigers, cheetahs and snow leopards are facing a severe threat of extinction for the above reasons. Efforts are being made through both *in situ* and *ex situ* conservation to protect these species from extinction. However, many felids reproduce poorly due to inappropriate captive management practices [1, 2]. Therefore, it is necessary to understand their endocrine status associated with ovarian activity, conception and pregnancy in order to identify optimal captive breeding conditions to facilitate conservation efforts [3].

Circulating steroid hormones reflect the reproductive status of animals [4], but the reliability of endocrine information may be compromised due to chemical immobilization or mechanical restraint during blood sampling. In the case of pregnancy diagnosis, immobilization includes an additional high risk of terminating a pregnancy due to stress [5, 6]. Non-invasive methods using urine and feces to assess the animal’s endocrine status have been established during the past few decades for a wide variety of species [7–9]. This technique uses analyses of urinary and fecal steroid metabolites for long-term assessment of reproductive capacity in males, of ovarian activity in females, and, in particular, for pregnancy diagnosis and parturition prediction in captive species [10–14]. Nowadays, non-invasive fecal hormone monitoring using testosterone, estrogen or progesterone metabolites has become a routine procedure in daily zoo management [3,13,15,16] for several mammalian species, including felids. Despite this widespread application, species-specific peculiarities in hormone metabolism, as well as in endocrine control mechanisms of reproduction, hinder rapid use of the measured hormone metabolites without additional biological validation.

The best example of the need for methodological validations is the lynx species, where fecal estrogens and gestagens do not reflect luteal activity during pregnancy. Both hormones rise steadily during pregnancy, and do not drop to basal levels at parturition but stay elevated throughout lactation and weaning [17, 18]. Furthermore, estrogen metabolites also do not reflect estrous activity in lynxes. These uncertain hormone profiles also do not allow discrimination between pregnancy and pseudopregnancies in the lynx, unlike in other felid species in which pseudopregnancies are characterized by a less steep and shorter increase in gestagen metabolites [10].

In felids, however, it is quite important to reliably diagnose pregnancy and, in particular, to distinguish it from a pseudopregnancy. Pseudopregnancies or non-pregnant luteal cycles occur in cat species after an infertile mating or early abortion. Induced ovulation leads to the formation of corpora lutea (CLs) which produce progesterone. At mid-pregnancy, feline CLs require extraovarian luteotropic signals which most likely come from the placenta. After parturition or a failed pregnancy (pseudopregnancy), the CLs undergo regression and cease progesterone production. Thus, as an alternative to progesterone, placental hormones are suitable to be used for pregnancy detection [19]. For several felid species, relaxin has been shown to be a reliable pregnancy marker during mid-pregnancy [20–22] and can be detected in urine after substantial ultrafiltration [23]. The main disadvantage of relaxin is its pregnancy-related time course, being highest at mid-pregnancy and falling to baseline during the last trimester, and hence, prediction of parturition appears unachievable with this approach.

Another type of placental hormone—the prostaglandins—are also indicators of pregnancy, and metabolites of this hormone class are also detectable in urine and feces [24]. Prostaglandins are involved in regulating ovarian, uterine and placental functions [25]. Placental prostaglandin F2α (PGF2α) acts as a luteolytic agent in domestic cats [26] and in ruminants [27, 28]. In cattle, uterine PGF2α plays an important role in the cyclic regression of CLs [29, 30]. PGF2α is metabolized within minutes to PGFM (13,14-dihydro-15-keto-PGF2α) in the lungs [31] which has a longer half-life in blood than its original compound [32]. In carnivores, PGFM has been applied as a useful analytical marker of PGF2α [33, 34] and in dogs PGFM is elevated just 2–4 days prior to parturition [34]. In many felid species, however, this prepartal PGFM increase in urine and feces occurs several weeks before parturition, which thus indicates PGFM as a clear signal differentiating between pregnant and pseudopregnant luteal cycles in domestic and nondomestic felid species [35–37]. However, big cats from the panthera lineage seem to behave differently from any other lineage of the Felidae family. In particular, measurements of fecal PGFM in an Indochinese tiger (*Panthera tigris corbetti*) and a Persian leopard (*Panthera pardus ciscaucasica*) revealed quite low levels of this pregnancy marker during the last trimester of pregnancy and the absence of a characteristic peak around parturition [36]. We conclude that more samples collected during a time course from individual cats of the panthera linage are needed to conclusively establish the suitability of the PGFM pregnancy test in big cats.

Therefore, the objectives of our study were (1) to assess the reliability of PGFM as a marker for pregnancy detection in big cats based on additional samples from pregnant female Asiatic lions, Bengal tigers and jaguars with average gestation periods lasting 107, 102 and 98 days respectively [13], (2) to identify the predominant fecal metabolites derived from prostaglandins, and (3) to compare patterns of immunoreactive PGFM metabolites among three species of the panthera lineage.

## Materials and Methods

### Animals and sampling

Between 1–4 fecal samples from known pregnant Asiatic lions (n = 2), Bengal tigers (n = 4) and jaguars (n = 2) (a total of 217 samples) were collected weekly (except over a few weeks in Asiatic lions and tigers) over a period of 18 (lion and tiger) and 17 weeks (jaguar), also including several samples following parturitions. Samples were collected in the morning between 8 and 9 am, without urine contamination, at the Nehru Zoological Park (NZP), Hyderabad. Approximately 10 g of fecal samples were collected and stored in a freezer (-20°C) until further processing. Information on mating and parturition were collected regularly from Nehru Zoological Park during the study period. Permission to collect fecal samples from Hyderabad Zoo was granted by the Director of Zoos, Andhra Pradesh, based on a memorandum signed between CCMB and Hyderabad dated 30/08/1999.

### Extraction of fecal samples

Fecal samples were processed as described previously [10, 13]. Briefly, fecal samples were dried, pulverized and 0.2g of fecal powder was boiled in 5ml of 90% ethanol for 20 min. The samples were centrifuged at 500g for 20 min, the supernatant was recovered and the pellet was resuspended in 5ml of 90% ethanol before re-centrifuging. The supernatants were pooled, dried in an oven and re-dissolved in 1ml of 100% methanol. The fecal extracts were stored at -20°C until assayed. For comparison of extraction procedures, 11 samples collected from pregnant females of 3 felid species (lynx, cheetah and leopard) were extracted according to the procedure described above and the standard procedure used in the laboratory in Berlin by extracting 0.5 g of wet feces for 30 min by shaking with 4.5 ml of 90% methanol, followed by centrifugation (15 min at 1200 g) and transferring the supernatant into a new tube. We did not find any significant differences in concentrations between these two extraction methods (M-W U = 40, P = 0.068).

For HPLC analysis, 1ml of fecal extract was purified in octadecyl C18 columns (200 mg ecf-C18 Sap columns, 0.5 mL; Macherey-Nagel, Düren, Germany) which were equilibrated with 2ml of 100% methanol followed by 20mM of Tris buffer plus 10% methanol. The columns were washed with 4ml of 20mM Tris buffer plus 10% methanol and the elution was carried out in 100% methanol. The eluates were evaporated in a heater at 55°C under nitrogen and dissolved in 225 μL 40% methanol. This experiment was carried out at the Leibniz Institute for Zoo and Wildlife Research in Berlin, Germany.

### PGFM antibody

The PGFM antibody was generated in rabbits against 9α,11α-dihydroxy-15-oxo-prost-5-en-1-oic acid-BSA as described by Schlegel, et al. [38]. The antibody was characterized by a high specificity toward PGFM (100%), low binding to PGEM (1.9%) and PGF2α (0.5%), and negligible cross-reactivities (<0.1%) to tetranor-PGFM, tetranor-PGEM, 11-PGF2α, PGF2β, PGE and PGAM [36].

### EIA procedure

The EIA was performed as previously described by Finkenwirth et al. [35]. Briefly, a 96 well microtiter plate was coated with affinity purified goat anti-rabbit IgG antibody and duplicates of 20 μl standards (0.4–400 μg/well) and fecal extracts, prepared in assay buffer (50 mM Na_2_HPO_4_/Na_2_HPO_4_, 0.15 M NaCl, 0.1% BSA, pH 7.4), were added simultaneously into the respective wells along with 100 μl of PGFM-HRP (horse radish peroxidase) conjugate which was diluted in assay buffer (1:20,000). Then, 100 μl of PGFM-specific antiserum (diluted 1:20,000 in assay buffer) was added to all the wells, except the blank, and the plates were incubated overnight at 4°C. The plates were washed the following day with washing solution. Thereafter, 150 μl of substrate solution (1.2 mM H_2_O_2_, 0.4 mM 3,3’,5,5’-tetramethylbenzidine in 10 mM sodium acetate, pH 5.5) was added to each well and incubated in the dark. The reaction was stopped with 50 μl of 4N H_2_SO_4_ and the color[*US English*] intensity was measured at 450 nm in an ELISA reader (Thermo Multiskan Spectrum Plate Reader, version 2.4.2, Thermo Scientific, Finland.

### High pressure liquid chromatography

High-pressure liquid chromatography was used to characterize immunoreactive fecal prostaglandin metabolites in samples from two Bengal tigers (days 89, 97, 98, 100, and days 89, 97, 98, 100 of pregnancy, respectively) and one jaguar (days 70, 73, 76, and 77 of pregnancy). Separation of analytes was performed according to our standard protocol [35]. Aliquots of purified fecal extracts (100 μL) were separated on a reversed-phase Ultrasep ES100/RP-18/6 mm HPLC column (150 x 4 mm; Sepserv, Berlin, Germany). The mobile phase consisted of a mixture of water and acetic acid in the ratio 100:0.02 (v:v), and acetonitrile and acetic acid in the ratio 100:0.02 (v:v). The following gradient of both diluents was performed: 20:80 (v:v) at 0 min, linear gradient to 30:70 (v:v) at 5 min, linear gradient to 70:30 (v:v) at 15 min, maintenance of 70:30 (v:v) until 20 min and return to the initial condition of 20:80 (v:v) at 21 min in preparation for the next injection. The flow rate was set at 1 mL/min and fractions of 0.33 mL were collected at 20-sec intervals over a period of 20 min. The elution positions of standards had been determined in separate HPLC runs after injection of 500 ng PGFM, tetranor-PGFM (Cayman Europe, Tallinn, Estonia) and PGF2α (Sigma Chemie GmbH, Deisenhofen, Germany). Aliquots of 10 μl of each HPLC fraction were subjected to liquid chromatography–mass spectrometry (LCMS) analyses. To generate the HPLC immunograms, the remaining fraction was lyophilized, reconstituted in 100 μL of 40% methanol, and subjected to the PGFM EIA as described above.

### Liquid chromatography–mass spectrometry

Mass spectrometry was carried out on an API3200 QTrap LC/MS/MS system with a Turbolon Spray electrospray ionization (ESI) source (AB SCIEX, Palo Alto, CA, USA) using multiple reactions monitoring and equipped with a Z-spray ESI interface as described earlier [35]. Chromatographic separation was achieved at 30°C on a Gemini C18 column (50 x 4 mm, 3 mm; Phenomenex, Torrance, CA, USA), where the injection volume was 20 μL and the mobile phase consisted of water and acetonitrile and formic acid in the ratio 80:20:0.1 (v:v:v) with a flow rate set to 0.5 mL/min. In the selected reaction-monitoring mode, the instrument monitored the m/z 353 to 113 (PGFM), 311 to 121 (tetranor-PGFM), and 353 to 309 (PGF2α).

To confirm PGFM and to identify other metabolites in fecal extracts detected by the PGFM antibody, 20 μL of the immunoreactive HPLC fractions were subjected to LCMS analysis. The presence of three transitions with a signal-to-noise ratio above three associated to the expected retention time allows identification of the analyte.

### Statistical analysis

For all animals the values (ng/g fecal dry weight) are represented as means ± standard error of the mean (SEM). Because the frequency of sample collection varied, weekly means were calculated for all the individuals and this was plotted together with the SEM in the graph. Non-pregnant samples were unavailable, hence, samples from before mating and after parturition were considered, to calculate the baseline PGFM levels in non-pregnant animals. The Mann–Whitney U test (M–W test) was used for testing differences in PGFM levels between weeks and terms. To examine differences in PGFM concentrations, the entire pregnancy period was divided into three equal periods as early (1–5 weeks), mid (6–10 weeks) and late term (11–15 weeks) for Asiatic lion, Bengal tiger and jaguar. We used the Friedman nonparametric repeated measures ANOVA to check for differences of fecal PGFM concentrations between the terms. All statistical analyses were carried out using SPSS 17.0.

## Results

### HPLC analysis of PGFM and immunoreactive eicosanoids in fecal extracts

**Fig 1:**
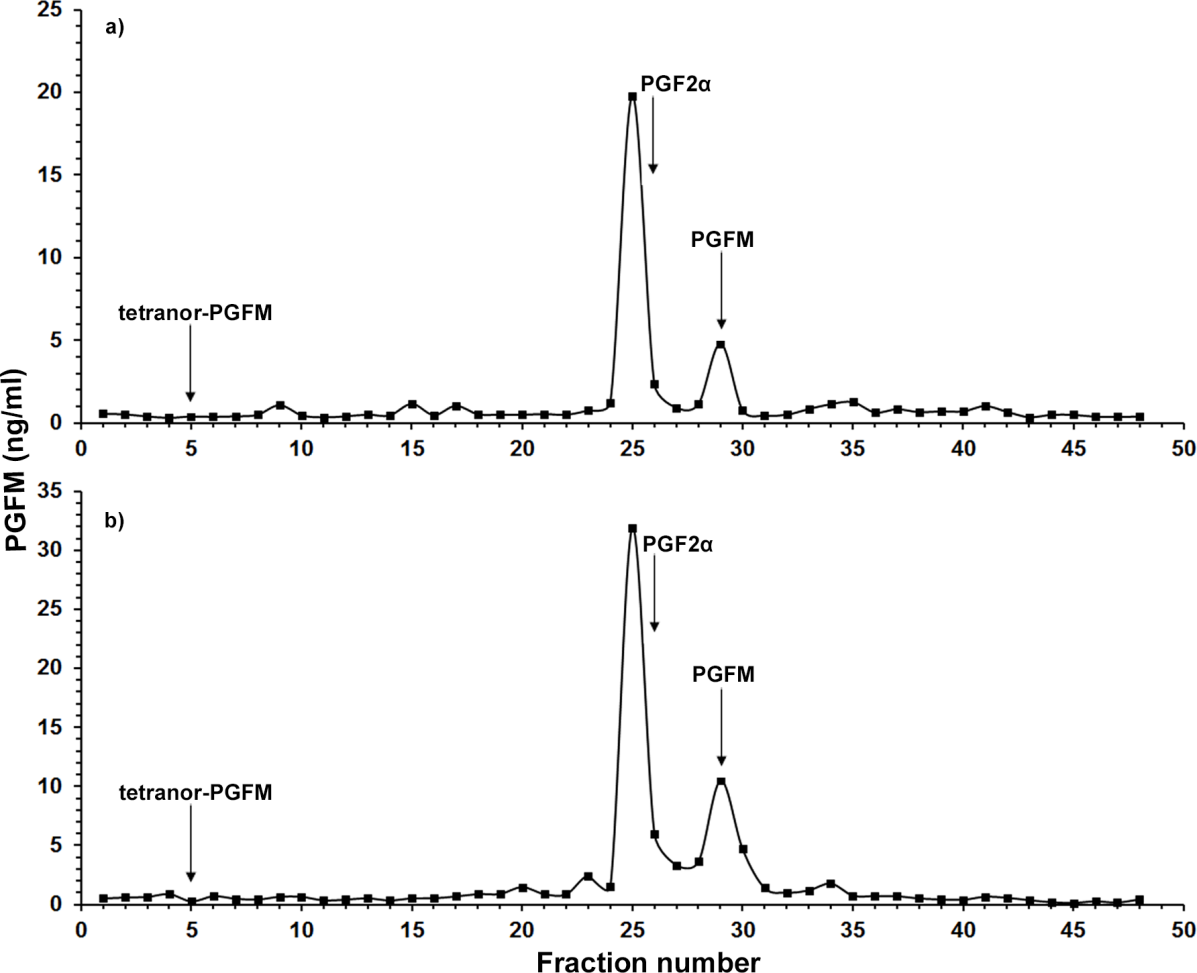
HPLC elution profile of immunoreactive PGFM in fecal extracts of a pregnant tiger (days 97, a) and a pregnant jaguar (days 76, b). Fecal extracts of each animal were separated by RP-HPLC and then immunoreactivitiy of each fraction was determined by the PGFM EIA. Arrows indicate the elution positions of prostaglandin standards.

The HPLC immunoreactive chromatogram (Fig 1A) shows two elution peaks at fraction number 25 (close to the PGF2α standard in fraction 26) and number 29 in a pregnant tiger’s fecal samples (days 97). LC-MS analysis confirmed the presence of PGFM in fraction 29 based on its specific transitions, whereas PGF2α could not be identified either in fraction 25 or in fraction 26. Despite its cross-reactivity with the PGFM antibody, the immunoreactivity in fraction 25 did not co-elute with any of the prostaglandin standards available in the laboratory (data not shown) and is classified as “unknown metabolite”. In addition, our antibody did not show cross-reactivity toward PGF2α, so the presence of PGF2α can be definitely excluded. A similar HPLC immunoreactivity profile was observed in jaguar fecal extracts from a sample collected on Day 76 of pregnancy (Fig 1B). As in the tiger, the immunoreactivity in fraction 29 corresponds to the position of standard PGFM which was identified by LC-MS whereas the major peak in fraction 25 remained unidentified.

**Fig 2:**
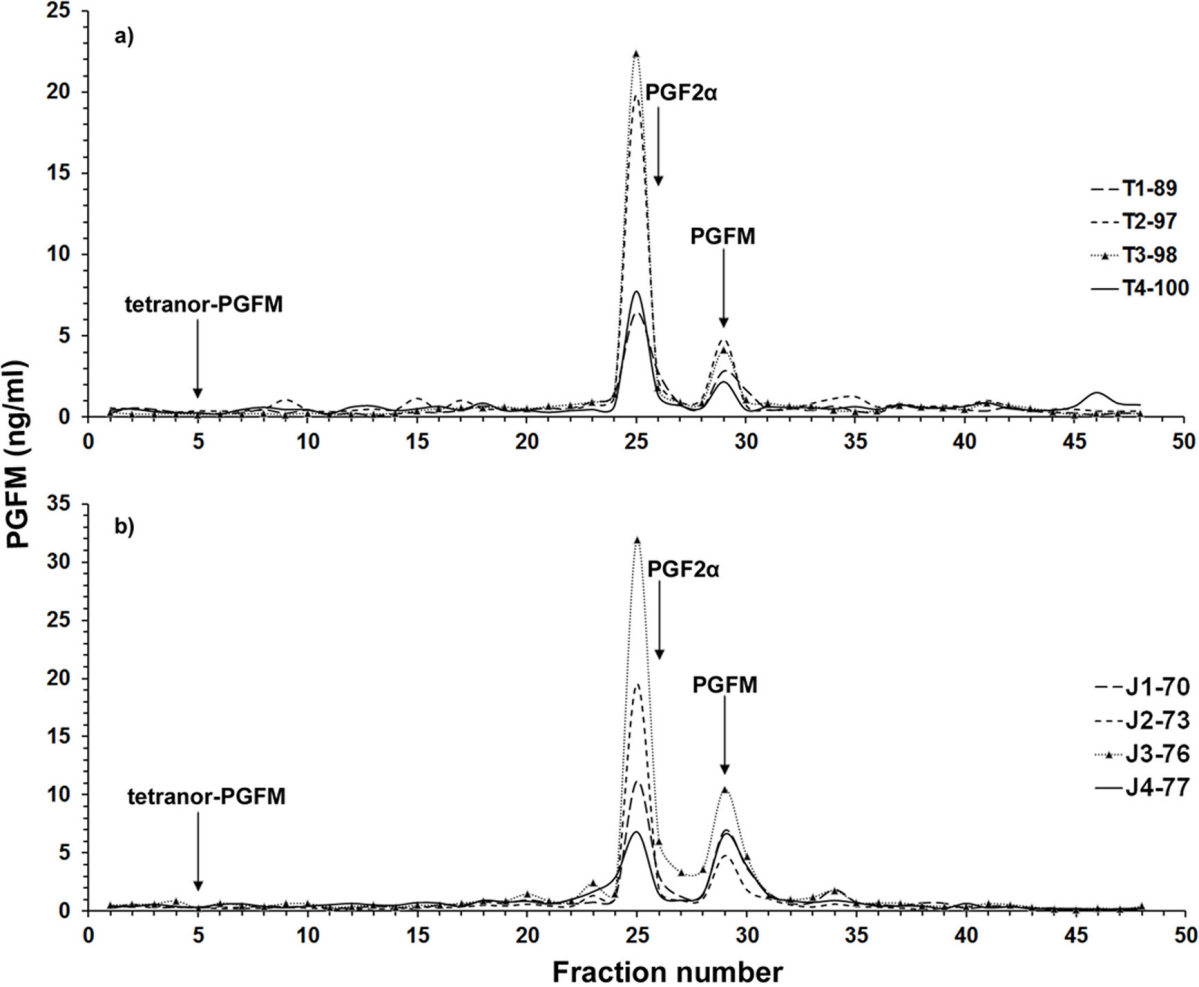
HPLC elution profiles of four fecal extracts from a Bengal tiger collected on Days 89, 97, 98 and 100 (a) and from a jaguar collected on Days 70, 73, 76 and 77 (b). Immunoreactivitiy of each fraction was determined in the PGFM EIA, arrows represent the elution positions of reference standards.

A comparison of the immunoreactivities in pregnant samples between days 89 and 100 in tiger and between 70 and 77 in jaguar revealed PGFM as a minor portion whereas the major portion consists of the unknown metabolite in fraction 25 varying distinctly between different days of pregnancy (Fig 2A and 2B). No tendency for changes in composition became detectable with approaching parturition.

### PGFM profile in feces of pregnant lions, tigers, and jaguars

**Fig 3:**
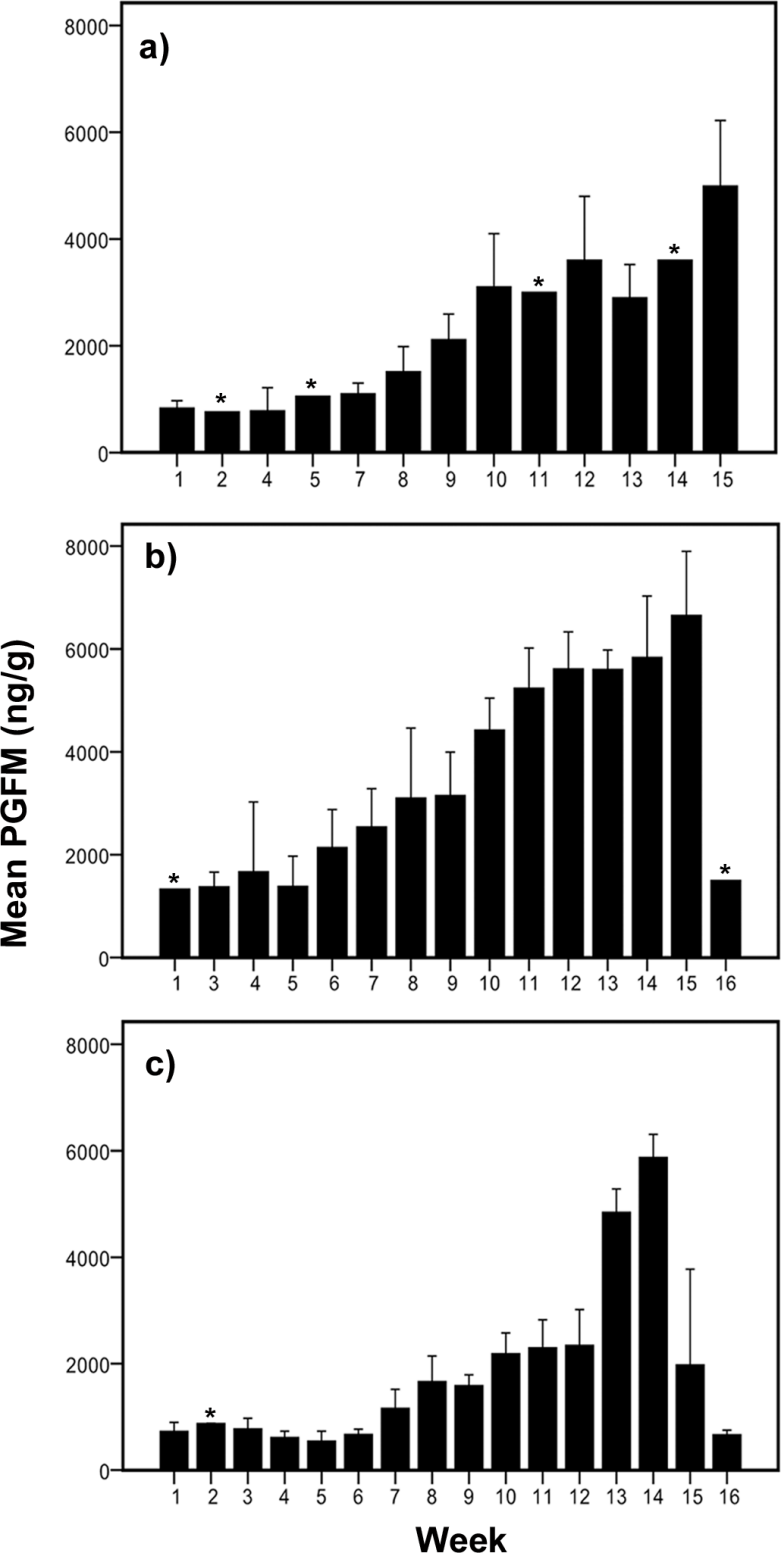
Fecal PGFM concentrations measured by EIA in samples of two pregnant lions (a), four pregnant tigers (b) and two pregnant jaguars (c), given as weekly means ± SEM. Asterisks indicate single sample per week.

**Fig 4:**
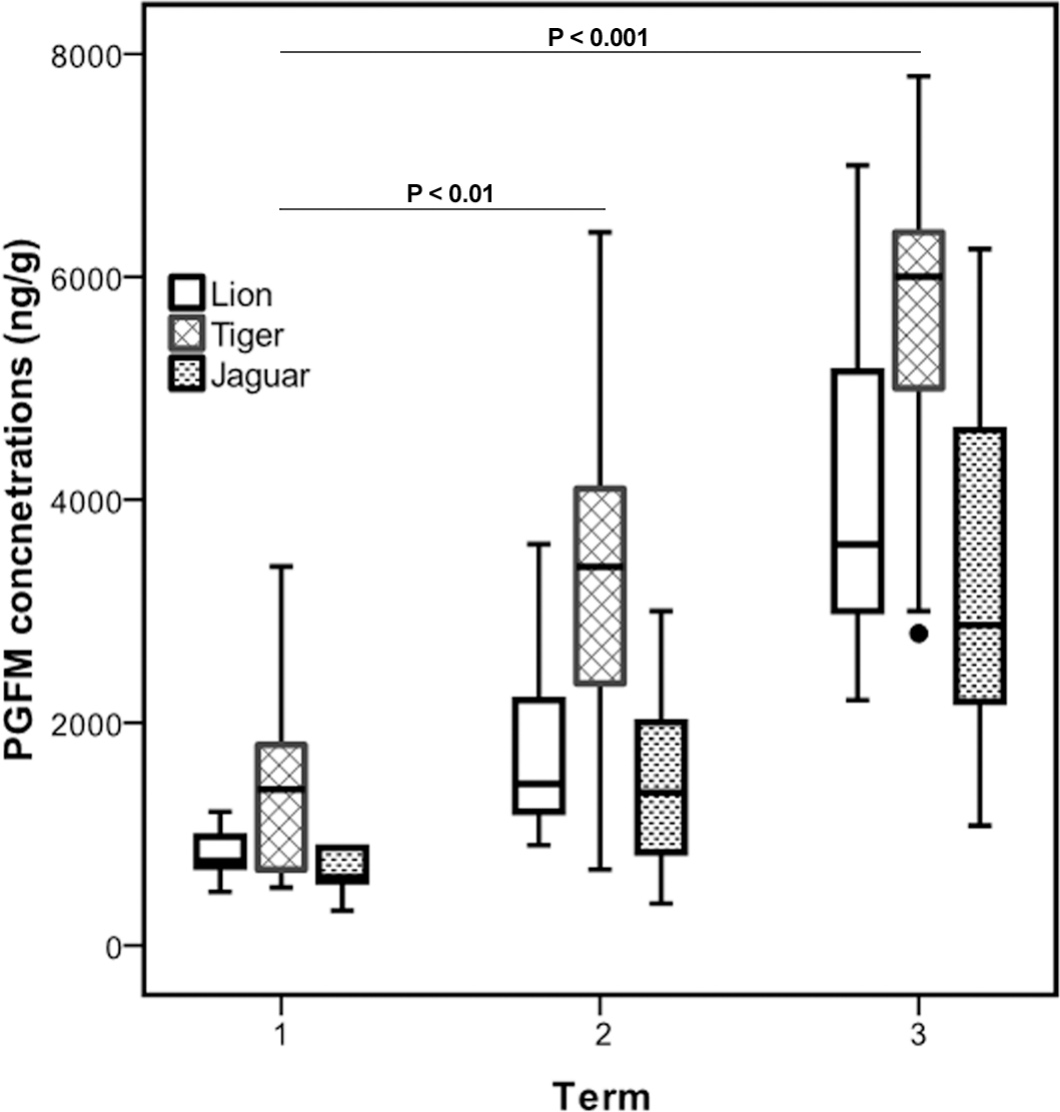
Term-wise PGFM concentration measured by EIA in Asiatic lion, Bengal tiger and jaguar. To examine differences in PGFM concentrations between different gestation periods, we divided pregnancy into three equal periods as early (1–5 weeks), mid (6–10 weeks) and late term (11–15 weeks) for Asiatic lion, Bengal tiger and jaguar.

Analyses of PGFM were carried out in samples from two pregnant lions, four pregnant tigers and two pregnant jaguars. Fig 3A depicts the weekly (1–4 samples /week, mean ± SEM) PGFM concentrations generated from the two Asiatic lions which varied significantly among the weeks (Friedman test χ^2^ = 18.00, P = 0.006). The mean basal PGFM levels during the first 7 weeks following conception ranged between 830 and 1100 ng/g. Further, PGFM concentrations started to increase from week 8 onwards (1512 ± 236 ng/g) until parturition (4987 ± 615 ng/g). Compared to basal concentrations (830–1100 ng/g), this increase in PGFM concentration was significant from week 9 onwards (P<0.05). Altogether, PGFM concentrations varied significantly among early, mid and last term of pregnancy (Friedman test, χ^2^ = 13.56, df = 2, P = 0.001; Fig 4).and increased significantly from early to last term of pregnancy (M-W test, P<0.001).

A similar profile was obtained from the 4 pregnant tigers (Fig 3B). Baseline levels of PGFM ranged from 1133 to 1665 ng/g during the first 5 weeks of pregnancy, then rose significantly after the 9^th^ week of pregnancy, remained elevated during the course of pregnancy and peaked the week prior to parturition (6650 ± 623.8 ng/g). Thereafter, PGFM concentrations dropped to baseline levels (1500 ng/g) the week following parturition. PGFM levels showed significant differences between the terms (Friedman test, χ^2^ = 30.12; df = 2, P = 0.001) and significant increases from the first to last term (M-W test P<0.001; Fig 4).

The course of PGFM from the two jaguars revealed a different pattern (Fig 3C). Mean basal levels of PGFM ranged between 546 to 875 ng/g during the first 6 weeks of pregnancy, showing steadily increasing concentrations beginning at week 7 until week 12 followed by a distinct increase towards peak levels the week prior to parturition (5875 ± 216.5 ng/g). Thereafter, PGFM dropped to basal levels. PGFM concentrations varied significantly between the first term (675.5 ± 42.1 ng/g) and the last term of pregnancy (3378 ± 346.2 ng/g, Friedman test, χ^2^ = 30.47, df = 2, P = 0.001, Fig 4).

## Discussion

Non-invasive methods using urine and feces have been used to detect pregnancy in many species by determining the levels of pregnancy-specific hormones. Elevated levels of progesterone (P4) and its metabolites (5α-pregnan-3α-ol-20-one) can be used for pregnancy detection in several species of felids [10, 13]. However, these hormones remain elevated even during pseudo-pregnancy, thus hampering pregnancy diagnosis. Pseudo-pregnancies are characterized by a shorter duration of luteal gestagen production after infertile mating, lasting approximately one-half to two-thirds of gestation [10]. In the lynx, the fecal hormone profiles for pseudo-pregnancies and pregnancies are quite similar [17, 18]. Alternatively the Witness-Relaxin pregnancy test [20] has been used to detect pregnancy from the urine of Iberian lynx [23] between days 26 and 46 of pregnancy but this was not a reliable method due to false positive results in the event of abortions. Among all pregnancy-related hormones, PGFM has been found to be a reliable indicator of pregnancy in several felid species, whereas discrepancies were found among the big cats of the panthera lineage [36].

In this study, the fecal PGFM concentrations remained distinctly elevated during the last trimester (last 30 days) of pregnancy, peaking towards parturition in all animals studied, the pregnant lion, tiger and jaguar. Compared to previous results [36] in felids of the panthera lineage, e.g., the Sumatran tiger, the black panther and Chinese leopard showed elevated PGFM during the last weeks of pregnancy. Overall fecal PGFM concentrations in our study species were higher than in previously described felids [36], but at the same time we did not find any significant differences in concentrations between extraction methods used in this study (dry method) [10, 13] and a previous study (wet method) [35]. Thus, we assume that the concentration differences between the present and our previous data [36] might be related to species differences and individual variations.

The HPLC immmunograms of extracted fecal samples in the three felid species showed a similar pattern, consisting of only minor portions of authentic PGFM (confirmed by LCMS analyses) whereas the major portion consists of an unknown immunoreactive component. This fits with our earlier data from other felid species in which PGFM was detected in more or less significant proportions but turned out not to be the major proportion of immunoreactivities [37].

The comparison of HPLC immunograms generated from different days of pregnancy revealed a high intra-individual variation in immunoreactive prostaglandin metabolites, but there was no pattern of metabolite composition that could be related to the stage of gestation. Thus, changes in metabolite composition seemed to occur at random, which confirms our earlier results. We assume that these differences may reflect changes in intestinal eicosanoid metabolism [39]. However, in contrast to our previous study, we were unable to detect significant amounts of a polar immunoreactivity at the elution positions of t-PGFM (fraction 5).

Unfortunately, we were so far unable to identify the unknown metabolite in fraction 25. Previous studies on the metabolism of radiolabelled PGF2α in rabbits and sheep revealed that PGF2α and PGFM were undetectable but found other metabolites [40, 41]. A radiometabolism study in rats [42] reported that a large portion of eicosanoid metabolites was excreted with the urine and feces, with many of the fecal metabolites being similar to those in urine although some metabolites in feces were not found in urine. Based on the HPLC profile of fecal metabolites in this study and urinary metabolites in our previous study in lynx [35], we also assume that the major fecal and urinary metabolites are identical. The substance in Fraction 25 elutes one fraction apart from PGF2α. Even though PGF2α has been reported to occur in human urine [43], our PGFM antibody did not show any cross-reactivity toward PGF2α which can therefore be definitely excluded.

Based on the facts that similar prostaglandin metabolites might be excreted with urine and feces [42], whereas tetranor metabolites cannot be considered because they do not correspond to the elution position of our unknown major metabolite, we suggest dinor metabolites as possible candidates. Urinary dinor metabolites have been reported to be useful indicators to accurately evaluate total body production of 8-iso-PGF2a [44].

Irrespective of the chemical character of the unknown major PGF2α metabolite in feces, our results confirm that the PGFM test can be used in big cats for pregnancy diagnosis. In all three *panthera* species analyzed here, an elevation of fecal PGFM metabolites over a threshold level of 1.5 μg/g dry feces can serve as pregnancy sign, allowing diagnosis with very few samples if not just a single one. This is a major advantage of PGFM pregnancy diagnosis in a captive animal setting, when regular fecal sample collection is unattainable. In such a setting, the fecal PGFM test is superior to the use of fecal gestagen assays. Another management tool in high demand is the prediction of parturition. Our data suggest that elevations in PGFM over 5 μg/g feces might be indicative for delivery within a week. However, drying fecal samples, shipping samples overnight to a laboratory, and performing the EIA generates a time lapse until the availability of results making this type of parturition prediction for now less feasible. Although this conclusion or application recommendation remains speculative until it has been validated on more individual profiles in different zoos.
